# A randomized controlled clinical trial of topical insulin-like growth factor-1 therapy for sudden deafness refractory to systemic corticosteroid treatment

**DOI:** 10.1186/s12916-014-0219-x

**Published:** 2014-11-19

**Authors:** Takayuki Nakagawa, Kozo Kumakawa, Shin-ichi Usami, Naohito Hato, Keiji Tabuchi, Mariko Takahashi, Keizo Fujiwara, Akira Sasaki, Shizuo Komune, Tatsunori Sakamoto, Harukazu Hiraumi, Norio Yamamoto, Shiro Tanaka, Harue Tada, Michio Yamamoto, Atsushi Yonezawa, Toshiko Ito-Ihara, Takafumi Ikeda, Akira Shimizu, Yasuhiko Tabata, Juichi Ito

**Affiliations:** Department of Otolaryngology, Head and Neck Surgery, Graduate school of Medicine, Kyoto University, Kyoto, 606-8507 Japan; Department of Biomaterials, Field of Tissue Engineering, Institute for Frontier Medical Sciences, Kyoto University, Kyoto, 606-8397 Japan; Department of Data Science, Institute for Advancement of Clinical and Translational Science, Kyoto University, Kyoto, 606-8507 Japan; Department of Clinical Innovative Medicine, Institute for Advancement of Clinical and Translational Science, Kyoto University, Kyoto, 606-8507 Japan; Department of Experimental Therapeutics, Institute for Advancement of Clinical and Translational Science, Kyoto University, Kyoto, 606-8507 Japan; Department of Clinical Pharmacology and Therapeutics, Kyoto University Hospital, Kyoto, 606-8507 Japan; Department of Otolaryngology, Okinaka Memorial Institute for Medical Research, Toranomon Hospital, Tokyo, 105-8470 Japan; Department of Otorhinolaryngology, Shinshu University School of Medicine, Matsumoto, 390-8621 Japan; Department of Otolaryngology, Ehime University Graduate School of Medicine, Ehime, 791-0295 Japan; Department of Otolaryngology, Graduate School of Comprehensive Human Science, University of Tsukuba, 305-8575 Tsukuba, Japan; Department of Otolaryngology, Head and Neck Surgery, Nagoya City University Graduate School of Medical Sciences and Medical School, Nagoya, 467-8601 Japan; Department of Otolaryngology, Kobe City Medical Center General Hospital, Kobe, 650-0047 Japan; Department of Otorhinolaryngology, Hirosaki University Graduate School of Medicine, Hirosaki, 036-8562 Japan; Department of Otorhinolaryngology, Graduate School of Medical Sciences, Kyushu University, Fukuoka, 812-8582 Japan

**Keywords:** Dexamethasone, Drug delivery system, IGF-1, Local application, Sudden sensorineural hearing loss

## Abstract

**Background:**

To date, no therapeutic option has been established for sudden deafness refractory to systemic corticosteroids. This study aimed to examine the efficacy and safety of topical insulin-like growth factor-1 (IGF-1) therapy in comparison to intratympanic corticosteroid therapy.

**Methods:**

We randomly assigned patients with sudden deafness refractory to systemic corticosteroids to receive either gelatin hydrogels impregnated with IGF-1 in the middle ear (62 patients) or four intratympanic injections with dexamethasone (Dex; 58 patients). The primary outcome was the proportion of patients showing hearing improvement (10 decibels or greater in pure-tone average hearing thresholds) 8 weeks after treatment. The secondary outcomes included the change in pure-tone average hearing thresholds over time and the incidence of adverse events.

**Results:**

In the IGF-1 group, 66.7% (95% confidence interval [CI], 52.9–78.6%) of the patients showed hearing improvement compared to 53.6% (95% CI, 39.7–67.0%) of the patients in the Dex group (*P* = 0.109). The difference in changes in pure-tone average hearing thresholds over time between the two treatments was statistically significant (*P* = 0.003). No serious adverse events were observed in either treatment group. Tympanic membrane perforation did not persist in any patient in the IGF-1 group, but did persist in 15.5% (95% CI, 7.3–27.4%) of the patients in the Dex group (*P* = 0.001).

**Conclusions:**

The positive effect of topical IGF-1 application on hearing levels and its favorable safety profile suggest utility for topical IGF-1 therapy in patients with sudden deafness.

**Trial registration:**

UMIN Clinical Trials Registry Number UMIN000004366, October 30^th^, 2010.

## Background

Sudden sensorineural hearing loss (SSHL), an unexplained unilateral hearing loss with an onset of less than 72 h, is a common disease with acute onset hearing impairment. The incidence of SSHL is reportedly 5 to 20 patients per 100,000 persons per year [1]. Approximately 35,000 patients with SSHL consult a doctor each year in Japan [2]. The standard treatment for SSHL is systemic corticosteroid treatment [3,4]. Hearing improvement after systemic corticosteroids occurs in 50% of the patients, but approximately 20% of the patients show no response [5]. Further, systemic corticosteroid treatment often causes adverse events [6] that can occasionally be life-threatening [7]. As an alternative for systemic corticosteroids, intratympanic corticosteroid treatment by direct injection into the middle ear has recently gained wide popularity because of the low risk for systemic adverse events and because of the potential delivery of high concentrations of a corticosteroid into the inner ear [8]. Intratympanic corticosteroid therapy is commonly used for the treatment of SSHL, after the failure of systemic corticosteroid treatment [9-15]. However, the supporting evidence for its use is weak because of the limitation in the study design and power [16].

A major difficulty in treating sensorineural hearing loss is the poor regeneration capacity of the mammalian cochlea. Therefore, protecting the cochlea from irreversible degeneration is a practical strategy. Several growth factors have been investigated for their protective effects on the sensory hair cells of the cochlea [17,18]. The focus of this study was on insulin-like growth factor-1 (IGF-1), which has been used for the treatment of insulin-resistant diabetes and dwarfism. IGF-1 also plays crucial roles in both the development and maintenance of the cochlea [19,20]. We used a gelatin hydrogel, which enables the sustained release of proteins or peptides, for the delivery of IGF-1 into the cochlear fluid [21]. We previously performed a successful series of animal experiments using this method [21,22]. A prospective, single-armed clinical trial in patients with SSHL refractory to systemic corticosteroids was then performed, the results of which indicated the safety and efficacy of topical IGF-1 therapy in comparison to the historical control of hyperbaric oxygen therapy [23].

The goal of the current study was to investigate the efficacy and safety of topical IGF-1 therapy as a novel therapeutic option for SSHL. We conducted a multicenter, randomized clinical trial to compare topical IGF-1 therapy and intratympanic corticosteroid therapy for treating SSHL refractory to systemic corticosteroids.

## Methods

### Study design and patients

This was a multicenter, randomized, open, parallel-group trial. The study was conducted from November 2010 through October 2013 at 9 tertiary referral hospitals in Japan. The trial followed the guiding principles of the Declaration of Helsinki. The study protocol, manual of procedures, and informed consent form were approved by the institutional review boards of all participating sites (Ethical Committee of the Graduate School of Medicine, Kyoto University [C470], Ethical Committee of the Graduate School of Medicine, Hirosaki University [2011–145], Ethical Committee of University of Tsukuba Hospital [H23-13], Ethical Committee of Toranomon Hospital [2011-4-15], Ethical Committee of Shinshu University Hospital [1705], Ethical Committee of Nagoya City University Hospital [45-11-0005], Ethical Committee of Kobe City Medical Center General Hospital [1], Ethical Committee of Ehime University Hospital [1105003], and Ethical Committee of the Graduate School of Medicine, Kyushu University [23011]). All patients provided written informed consent. Eligible participants were all adults, 20 years or older, who had SSHL defined as a unilateral sensorineural hearing loss of at least 30 decibels (dB) sound pressure level (SPL) over at least three test frequencies that developed within 3 days. They also met the following eligibility criteria: they had been diagnosed as having SSHL within 25 days of onset; they presented with an abnormality in the distortion product of otoacoustic emissions; and they showed less than 30 dB hearing improvement in the mean hearing level, based on pure-tone audiometry (PTA) at five tested frequencies (0.25 kHz, 0.5 kHz, 1.0 kHz, 2.0 kHz, and 4.0 kHz) after more than 7 days of systemic corticosteroid treatment. Similar to our previous trial [23], we excluded patients with active chronic otitis media, acute otitis media, otitis media with effusion or dysfunction of the auditory tube, malignant tumors, and systemic diseases. All patients underwent imaging examinations to rule out retrocochlear pathology.

### Randomization and masking

Patients were randomly assigned (1:1) to receive either topical IGF-1 therapy or intratympanic dexamethasone (Dex) therapy. Randomization was performed centrally with stratification by the study sites and the mean hearing thresholds, based on the PTA at the five frequencies tested at registration (lower than 90 dB SPL vs. 90 dB SPL or higher). The randomization sequence was generated by a third-party contract clinical research organization (independent from the trial investigators). Local investigators used a web-based system during enrolment, which then automatically assigned patients to either treatment group. Besides central randomization, no further masking was used in this open-label study.

### Procedures

The treatment was performed within 7 days of enrolment and systemic corticosteroid treatment was completed by the time of enrolment. Gelatin hydrogels were produced from porcine skin gelatin (Nitta Gelatin Inc., Osaka, Japan), based on a previously described method [23,24]. Mecasermin (Somazon [10 mg for injection]; Astellas Pharma, Inc., Tokyo, Japan), a recombinant human IGF-1, was dissolved in physiological saline at a final concentration of 10 mg/mL. Sixty minutes before application, a 30 μL sample of mecasermin solution was mixed with 3 mg of gelatin hydrogel. After tympanostomy under local anesthesia with 1% lidocaine, the hydrogel (which contained 300 μg of mecasermin) was placed in the round window niche of the middle ear; a single application was used. The control group received four 0.5 mL doses containing 3.8 mg/mL dexamethasone sodium phosphate (Orgadrone injection [1.9 mg]; MSD, Inc., Tokyo, Japan) that was injected into the middle ear through the tympanic membrane. In the literature [9-15], a variety of regimens for intratympanic injections of corticosteroids have been used. Considering practical use in common clinical settings, we chose four doses. Injections were performed over 4 consecutive days in principle. Within at least 7 days, four injections were administered. Patients were placed in the supine position with the affected ear slightly raised and remained in this position for 30 min after the injection. For 16 weeks after treatment, the patients were examined at the outpatient clinics of the participating sites. The PTA was measured on the day of enrolment, and then at 1, 2, 4, 8, 12, and 16 weeks after treatment. During the observation period, which totaled 16 weeks, all adverse events were recorded.

### Outcomes

The primary outcome measure was the proportion of patients showing hearing improvement of 10 dB or greater in the mean hearing level. Hearing improvement was based on PTA at five tested frequencies and was defined as better than slight recovery, based on the criteria for hearing improvement determined by the Sudden Deafness Research Committee of the Japanese Ministry of Health, Labor and Welfare in 1984 (Table 1) at 8 weeks after treatment. Briefly, the complete recovery includes patients showing recovery of a hearing level within 20 dB SPL or to the similar level to the opposite side, the marked recovery is more than 30 dB recovery, and the no recovery is less recovery than 10 dB. Secondary outcome measures included the change in the pure-tone average hearing thresholds over time (i.e., from the first audiogram to the 16-week follow-up audiogram), the proportion of patients showing hearing improvement at 12 and 16 weeks after treatment, and the incidence of adverse events during the observation period. In addition to checking vital signs in the physical examination at each visit, laboratory studies were performed at registration, and then at 1 week, 8 weeks, and 16 weeks after treatment.

**Table 1:** **Criteria for hearing improvement determined by Sudden Deafness Research Committee of the Japanese Ministry of Health, Labour and Welfare in 1984**

Complete recovery	Recovery of a hearing level within 20 decibels (dB) at all five frequencies tested (0.25, 0.5, 1.0, 2.0 and 4.0 kHz) or recovery to the same level as the opposite side in pure-tone auditometry
Marked recovery	30 dB and over recovery in the mean hearing level at the five frequencies tested
Slight recovery	Recovery of better than 10 dB and less than 30 dB in the mean hearing level at the five frequencies tested
No response	Less recovery than 10 dB in the mean hearing level at the five frequencies tested

### Statistical analysis

The null hypothesis was that the effect of topical IGF-1 treatment on the proportion of patients showing hearing improvement would not be superior to intratympanic injection of Dex. The sample size was determined based on our previous findings and on published papers. In our previous clinical trial of topical IGF-1 therapy, no patient that had been enrolled later than 26 days after sudden hearing loss recovered their hearing [23]. The proportion of patients with hearing improvement after topical IGF-1 treatment was 62.5% (10 of 16 patients) at 12 weeks and 68.8% (11 of 16 patients) at 24 weeks, using the eligibility criteria of patients with SSHL within 25 days after onset of sudden hearing loss. Based on these findings, we hypothesized that the expected proportion of patients showing hearing improvement with topical IGF-1 therapy would be 65%. To determine the expected proportion of patients showing hearing improvement for intratympanic Dex therapy, we referred to the information in several publications [9-15] that were available in October 2010 and used the following criteria: i) the sample size had more than 10 participants and ii) intratympanic Dex therapy was a salvage treatment (Table 2). In these previous reports, the mean proportion of patients showing hearing improvement was 39% (range, 21–58%). Therefore, the proportion of patients who would show hearing improvement with intratympanic Dex therapy was expected to be approximately 40%. The sample size was based on the continuity-adjusted arcsine test with a one-sided significance level of 0.05 and a power of 0.80. The required sample size was 120 participants, assuming that 5% of the patients would be excluded from the analysis.

**Table 2:** **Previous studies of intratympanic dexamethasone therapy as a salvage treatment**

	**No. of patients**	**PTA recovery**	**Dexamethasone concentration**	**Doses**	**Proportion of recovery (%)**
Ho et al. [9]	15	28 dB	4 mg/mL	3 doses, once a week	53%
Choung et al. [10]	34	9 dB	5 mg/mL	4 doses, twice a week	39%
Roebuck et al. [11]	31	12 dB	24 mg/mL	Single	29%
Haynes et al. [12]	40	5 dB	24 mg/mL	Single	27.5%
Plontke et al. [13]	11	14 dB	4 mg/mL	Pump for 14 days	45%
Kakehata et al. [14]	24	17 dB	4 mg/mL	15 doses	58%
Lee et al. [15]	34	NA	4 mg/mL	6 doses for 14 days	21%
**Mean (95% CI)**	27 (21–33)				39% (30%–48%)

PTA, Pure-tone audiometry; NA, Not available; CI, Confidential interval.

All statistical analyses were performed on an intention-to-treat basis. The safety analyses were conducted on all patients who underwent randomization and received at least one dose of the study drugs. Efficacy was analyzed in all patients except those who had been excluded from the safety analyses due to eligibility violations. The differences between treatments in the proportion of patients showing hearing improvement at 8, 12, and 16 weeks after treatment were evaluated with Fisher’s exact test at a one-sided significance of 0.05. The difference between treatments in changes in pure-tone average hearing thresholds over time was investigated with repeated measures linear mixed model containing terms for treatment, time, and treatment-by-time interaction with an unstructured covariance structure [25]. The effect of treatment-by-treatment interaction was analyzed with the *t*-test at a one-sided significance of 0.05. The differences between treatments in the incidence of adverse events and in the baseline characteristics were analyzed with either Fisher’s exact test or the *t*-test at a two-sided significance of 0.05. Statistical analyses were performed by using SAS version 9.3 software (SAS Institute, Inc., Cary, NC, USA).

## Results

### Study overview

**Figure 1:**
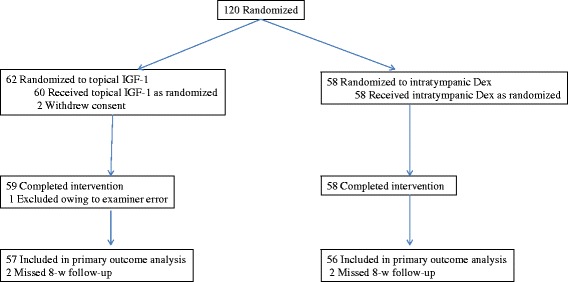
**Study overview.**

Patients from nine participating sites were enrolled between March 2011 and October 2013. The recruitment and enrollment period was originally planned to close in February 2013, but it was extended to meet the recruitment targets. There were 120 patients who consented to participate (Figure 1). All patients were randomized to either the IGF-1 group or the Dex group; 118 patients were included in the safety analysis (60 IGF-1, 58 Dex) because 2 patients from the IGF-1 group withdrew consent. Of the 118 patients who were included, 4 patients completed the treatments, but missed the 8-week follow-up (2 IGF-1, 2 Dex) and 1 patient was excluded owing to examiner error (1 IGF-1).

Baseline characteristics were comparable between the two treatment groups (Table 3). Between the IGF-1 and Dex groups, no significant baseline differences in demographics, physical characteristics, ear examination, or PTA thresholds were found except the proportion of patients presenting with aural fullness (Table 3). The mean age of all participants was 49.3 years, and 45.8% of the participants were male. The mean PTA thresholds in the affected and unaffected ears were 85.2 dB SPL (95% confidence interval [CI], 81.3–89.1) and 18.1 dB SPL (95% CI, 14.9–21.3), respectively. The mean number of days from symptom onset to study entry was 16.0 days (95% CI, 15.2–16.9). At enrolment, dizziness or vertigo was present in 45.8% of the patients, tinnitus was present in 84.8% of the patients, and aural fullness was present 64.4% of the patients.

**Table 3:** **Baseline characteristics of patients**

**Characteristics**	**Intratympanic Dex (n = 58)**	**Topical IGF-1 (n = 60)**	***P*** **value**
Age-yr, mean ± SD	50.1 ± 13.0	48.6 ± 14.0	0.557
Male sex-no. (%)	26 (44.8)	28 (46.7)	0.855
No. of days for study entry from onset, mean (95% CI)	16.3 (15.1–17.5)	15.8 (14.6–17.0)	0.574
Hearing improvement by pre-treatment-no. (%)			
>10 dB, <30 dB	13 (22.4)	17 (28.3)	0.528
<10 dB	45 (77.6)	43 (71.7)	
Hearing-dB pure-tone average, mean (95% CI)			
Affected ear	84.8 (79.1–90.4)	85.6 (80.0–91.2)	0.835
Unaffected ear	15.8 (12.4–19.2)	20.4 (15.0–25.7)	0.160
Other symptoms-no. (%)			
Dizziness/Vertigo	23 (39.7)	31 (51.7)	0.202
Tinnitus	49 (84.5)	51 (85)	>0.999
Aural fullness	44 (75.9)	32 (53.3)	0.013*

An asterisk indicates statistical significance with Fisher’s exact test. Dex: dexamethasone.

### Primary outcome

The primary outcome was the proportion of patients showing hearing improvement (10 dB or greater in pure-tone average hearing thresholds) at 8 weeks after treatment. In the Dex group, 53.6% (95% CI, 39.7–67.0%) of patients showed hearing improvement at 8 weeks after treatment, whereas in the IGF-1 group, 66.7% (95% CI, 52.9–78.6%) of patients showed hearing improvement (Table 4). The null hypothesis for the primary outcome was not rejected (*P* = 0.109). However, a trend was observed in the higher proportion of patients in the IGF-1 group showing complete or marked recovery (30 dB or greater in pure-tone average hearing thresholds) over that in the Dex group (Table 2).

**Table 4:** **Primary and secondary outcomes**

	**Intratympanic Dex**	**Topical IGF-1**	***P*** **value**
**Primary outcome**			
Proportion of patients showing hearing recovery at 8 weeks	53.6% (30/56)	66.7% (38/57)	0.109
	[95% CI: 39.7–67.0]	[95% CI: 52.9–8.6]	
Complete recovery	0.0% (0/56)	3.5% (2/57)	
Marked recovery	16.1% (9/56)	24.6% (14/57)	
Slight recovery	37.5% (21/56)	38.6% (22/57)	
No recovery	46.4% (26/56)	33.3% (19/57)	
**Secondary outcomes**			
Proportion of patients showing hearing recovery at 12 weeks	55.4% (31/56)	70.7% (41/58)	0.066
	[95% CI: 41.5–68.7]	[95% CI: 57.3–81.9]	
Complete recovery	0.0% (0/56)	5.2% (3/58)	
Marked recovery	21.4% (12/56)	31.0% (18/58)	
Slight recovery	33.9% (19/56)	34.5% (20/58)	
No recovery	44.6% (25/56)	29.3% (17/58)	
Proportion of patients showing hearing recovery at 16 weeks	54.7% (29/53)	67.9% (36/53)	0.116
	[95% CI: 40.4–68.4]	[95% CI: 53.7–80.1]	
Complete recovery	0.0% (0/53)	5.7% (3/53)	
Marked recovery	22.6% (12/53)	24.5% (13/53)	
Slight recovery	32.1% (17/53)	37.7% (20/53)	
No recovery	45.3% (24/53)	32.1% (17/53)	
**Adverse events**			
Serious	0.0% (0/58)	0.0% (0/59)	>0.999
Non-serious	43.1% (25/58)	35.0% (21/59)	0.452
Tympanic membrane perforation	15.5% (9/58)	0.0% (0/59)	0.001*
Otitis media	1.7% (1/58)	6.8% (4/59)	0.364
Otitis externa	0.0% (0/58)	1.7% (1/59)	>0.999
Tinnitus	8.6% (5/58)	0.0% (0/59)	0.027*
Nausea/Vomit	3.4% (2/58)	3.4% (2/59)	>0.999
Others	24.1% (14/58)	30.5% (18/59)	0.535

Asterisks indicate statistical significance with Fisher’s exact test.

### Secondary outcomes

**Figure 2:**
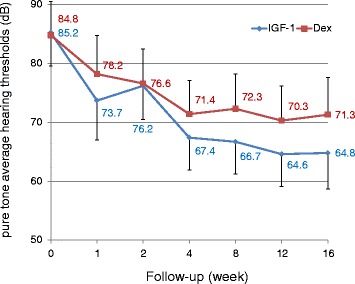
**Changes in pure-tone average hearing thresholds over time.** The difference between IGF-1 and dexamethasone (Dex) treatments in changes in pure-tone average hearing thresholds over time was statistically significant. Captions in the graph show mean values. Bars represent 95% confidence intervals.

The changes in the pure-tone average hearing thresholds occurring over time in both treatments are shown in Figure 2. The difference in the changes in the pure-tone average hearing thresholds over time between the treatments was statistically significant with repeated measures linear mixed model containing terms for treatment, time, and treatment-by-time interaction with an unstructured covariance structure (the effect for the interaction term [standard error]: −0.28 [0.10], *P* = 0.003). This demonstrated that pure-tone average hearing thresholds of the IGF-1 group significantly reduced over time, if changes in pure-tone average hearing thresholds over time in the Dex group were set to zero.

The proportions of patients showing hearing improvement (i.e., 10 dB or greater) at 12 weeks and 16 weeks after treatment were estimated as the secondary outcomes (Table 4). The null hypothesis for the proportions of patients showing hearing improvement at 12 weeks or 16 weeks after treatment was not rejected (*P* = 0.066 for 12 weeks; *P* = 0.116 for 16 weeks). At 12 and 16 weeks after treatment, there was a trend in the IGF-1 group showing a larger number of patients with complete and marked recovery when compared to the Dex group (Table 4).

### Adverse events

No serious adverse events were observed in either treatment group. During the observation period, moderate adverse events occurred in 43.1% (95% CI, 30.2–56.8) of the patients in the Dex group and in 35.0% (95% CI, 23.1–48.4) of the patients in the IGF-1 group (Table 4). No significant difference in the incidence of adverse events was found between treatments (*P* = 0.452). Most adverse events, such as otitis media, otitis externa, tinnitus, and nausea or vomiting disappeared during the observation period. However, tympanic membrane perforation persisted in 15.5% (95% CI, 7.3–27.4%) of the patients in the Dex group at the end of the observation period. On the other hand, no patient in the IGF-1 group showed residual perforation in the tympanic membrane. The difference in the incidence of tympanic membrane perforation was statistically significant (*P* = 0.001).

## Discussion

This is the first randomized controlled clinical trial to test the efficacy of a growth factor for the treatment of sensorineural hearing loss. In the current study, we locally applied IGF-1 to patients with SSHL refractory to systemic steroids. The null hypothesis of the primary outcome was that the proportion of patients showing hearing improvement after topical IGF-1 therapy would not be better than that after intratympanic Dex therapy. The null hypothesis was not rejected in the present study. The major reason for this is an unexpectedly high proportion of patients showing hearing improvement after intratympanic Dex therapy. The proportion of patients showing hearing improvement after topical IGF-1 therapy was 66.7 to 70.7%, which was nearly identical to our hypothesized value of 65%, whereas the proportion of patients showing hearing improvement after intratympanic Dex therapy (range, 53.6– 55.4%) was higher than our hypothesized value of 40%.

Although the null hypothesis for the primary outcome was not rejected, this randomized controlled trial showed significantly better recovery of pure-tone average thresholds over time in the IGF-1 group, compared to the Dex group. In addition, a trend that the proportion of patients in the IGF-1 group who showed complete or marked recovery was higher than that in the Dex group was observed at 8, 12, or 16 weeks after treatment. Complete recovery of hearing was observed only in the IGF-1 group. These findings strongly suggest the superior efficacy of topical IGF-1 therapy over intratympanic Dex therapy.

In the current study, we used intratympanic corticosteroid therapy as a control treatment because it has widely been accepted as a salvage treatment for SSHL refractory to systemic corticosteroids [9-15]. The current randomized study also provided evidence for the safety and efficacy of intratympanic Dex therapy for SSHL refractory to systemic corticosteroids. Similar to the results of previous studies [9-15], no serious adverse events occurred in the Dex patient group. However, tympanic membrane perforation persisted in 15.5% of these patients, while tympanic membrane perforation was absent in the IGF-1 patient group. The incidence of tympanic membrane perforation in the Dex group in the present study was higher than the 3.9% incidence in a previous randomized trial of intratympanic corticosteroid therapy as the initial treatment [8]. This may be because of a difference in either the application regimen or the influence of the preceding systemic corticosteroid treatment. It is important to note that intratympanic injection of corticosteroids causes tympanic membrane perforation in a certain proportion of treated patients, while both our previous [23] and present results demonstrated no occurrence of residual perforation in tympanic membranes of patients treated with topical IGF-1 therapy. This indicated the superior safety of topical IGF-1 therapy over intratympanic Dex therapy. On the other hand, the high incidence of tympanic membrane perforation in the Dex group might affect hearing recovery outcomes in the Dex group.

The present findings indicate the efficacy and safety of topical IGF-1 therapy for SSHL. However, topical IGF-1 therapy requires surgical procedures and causes uncomfortable symptoms associated with the local application. In addition, spontaneous recovery of hearing occurs in 30 to 60% of patients with SSHL [5,26-28]. Therefore, with the need for balancing the harmful side effects and the benefits, SSHL patients showing insufficient hearing improvement after the administration of oral corticosteroids or after strict observation for 7 days may be good candidates for topical IGF-1 therapy. On the other hand, IGF-1 is a promoter of cell proliferation in some cellular contents. Therefore, a long-term follow-up of patients may be required. Of note, in our previous clinical trial, we locally applied IGF-1 in the middle ear of 25 patients with refractory SSHL [23]; in the 5-year follow-up, no tumor formation was identified in those patients.

## Conclusions

We performed a randomized, controlled clinical trial of topical IGF-1 therapy in patients with SSHL refractory to systemic corticosteroids and compared this treatment to intratympanic corticosteroid therapy. Present results suggest the possibility that IGF-1 is superior to intratympanic Dex therapy, but the current study design failed to confirm this possibility. The positive effect of topical IGF-1 application on hearing levels and its favorable safety profile suggest utility for topical IGF-1 therapy as a salvage treatment for SSHL.

## Abbreviations

CI: Confidence interval
dB: Decibels
Dex: Dexamethasone
IGF-1: Insulin-like growth factor-1
PTA: Pure-tone audiometry
SSHL: Sudden sensorineural hearing loss
